# Knowledge and Awareness of Glaucoma Among People Living in Taif City in the Western Region of Saudi Arabia

**DOI:** 10.7759/cureus.47728

**Published:** 2023-10-26

**Authors:** Fahad M Albaqami, Abdulaziz Saud Aljuaid, Waleed Khalid Alrabie, Muath Abdulrahim Alotaibi, Majed M Albaqami, Faisal Sultan Alharthi, Abdulhamid Alghamdi

**Affiliations:** 1 Ophthalmology, King Abdulaziz Specialist Hospital, Taif, SAU; 2 General Surgery, King Faisal Medical Complex, Taif, SAU; 3 General Practice, Ministry of Health, Taif, SAU; 4 Plastic Surgery, King Faisal Medical Complex, Taif, SAU; 5 General Practice, Ministry of Defense, Taif, SAU; 6 Ophthalmology, Taif University, Taif, SAU

**Keywords:** saudi arabia, awareness, risk factors, causes, glaucoma

## Abstract

Background

Glaucoma represents a significant global health challenge, characterized by progressive and irreversible optic nerve damage, visual field impairment, and potential blindness. Limited awareness can lead to delayed diagnosis and increased healthcare burden. This research explored glaucoma awareness in Taif City, Saudi Arabia. It delved into the influence of demographics, socioeconomic factors, and cultural beliefs on awareness. The study aimed to inform tailored awareness campaigns and policy decision-makers about regional awareness, ultimately contributing to effective healthcare initiatives in the region.

Methodology

A cross-sectional study was conducted in Taif City including adults (>18 years) using a community-based approach. Data were collected using an electronic questionnaire.

Results

This study included 1,000 participants. Most participants were males, aged 18-29 years, with 51.1% (n = 511) having at least a bachelor’s education. The media (n = 341, 34.1%) and relatives/friends (n = 336, 33.6%) were common sources of glaucoma information. Among all participants, 58.5% (n = 585) had heard of glaucoma, and many were uncertain about glaucoma’s definition and normal eye pressure values. About 63.1% (n = 631) believed in a cure, and 43.1% thought vision loss could be restored. Gender, education, and family history significantly influenced awareness. Closed-angle glaucoma (n = 297, 29.7%) and open-angle glaucoma (n = 231, 23.1%) were recognized types, with surgery (n = 371, 37.1%) and laser treatment (n = 274, 27.4%) perceived as potential cures.

Conclusions

This study revealed glaucoma awareness among adults in Taif City. Gender, education, and family history played significant roles in shaping awareness levels. There is a need for targeted educational efforts to improve knowledge about glaucoma in the community.

## Introduction

Glaucoma, an intricate group of ocular disorders, represents a significant global health challenge, characterized by progressive and irreversible optic nerve damage, visual field impairment, and potential blindness [1]. Glaucoma, a silent but devastating eye disease, poses a substantial threat to global eye health [2]. Its multifactorial etiology encompasses variables such as genetics, age, intraocular pressure, family history, and race [3]. Swift diagnosis, timely intervention, and effective management stand as pivotal factors in mitigating irreversible vision loss caused by this insidious condition [4].

Global disparities in glaucoma awareness are a pressing concern, with significant differences observed among various geographical regions and populations [5]. It is crucial to emphasize the importance of heightened awareness, especially with the given asymptomatic nature of the disease. Soqia et al. (2023) conducted a comprehensive study that shed light on the widespread lack of awareness, which, in turn, contributed to the alarming prevalence of undiagnosed glaucoma cases around the world [6].

This research initiative embarks on a comprehensive exploration of the depth and scope of glaucoma awareness within the intricate tapestry of Taif City. Nestled in the heart of western Saudi Arabia, this urban center encapsulates a dynamic amalgamation of individuals, each contributing a distinctive set of perspectives, beliefs, and lifestyles influenced by health literacy [7,8], social media platforms [9], educational backgrounds [10], cultural beliefs [11], and access to healthcare resources [12,13].

The investigation of glaucoma awareness in this context becomes an essential endeavor to address potential disparities in knowledge dissemination and to chart a course for targeted health education initiatives. Further, a critical aspect of glaucoma awareness research involves evaluating the efficacy of the ongoing awareness campaigns [14]. Assessing the outcomes of previous initiatives, such as those which were conducted by Saudi organizations such as the Saudi Glaucoma Society, can provide insights into which approaches have worked and which aspects need refinement.

In Taif, like many other places, limited knowledge about glaucoma’s subtle progression and potentially irreversible damage hinders early diagnosis, adequate management, and treatment initiation [15,16]. These intricate variables collectively impact the awareness landscape regarding glaucoma and its underlying risk factors [17]. Addressing these awareness gaps can significantly impact the management of glaucoma and potentially prevent irreversible vision loss.

Hence, this study aimed to assess the current level of awareness and knowledge of glaucoma; identify the main sources of information about glaucoma; determine the accuracy of understanding regarding the definition, symptoms, and risk factors of glaucoma; and examine the perception of glaucoma as a treatable and preventable condition. In addition, this study also aimed to analyze any associations between demographic factors (such as age, gender, and education level) and glaucoma awareness among the Taif City population.

The significance of this research extends beyond mere statistical enumeration. By scrutinizing not only the percentage of individuals cognizant of glaucoma but also delving into the nuanced fabric of understanding, misconceptions, and attitudes that underlie this awareness, the study endeavors to construct a comprehensive narrative. By painting a comprehensive portrait of glaucoma awareness within Taif City [18], the study not only contributes to the scientific literature but also empowers healthcare practitioners, policymakers, and educators with the insights required to curate effective and culturally sensitive awareness initiatives.

## Materials and methods

Study design and location

A cross-sectional study was conducted in Taif City adopting a community-based approach.

Inclusion and exclusion criteria

The study included both male and female adults (>18 years old) residing in Taif City. Individuals below 18 years of age and those who did not reside in Taif City were excluded from the study.

Sample size

To ensure the reliability of the study outcomes, the sample size necessary for investigating glaucoma awareness in Taif City was determined using the EPI Info program. Employing a 95% confidence level and a 5% margin of error, around 384 participants were required. By considering the likelihood of a 10% non-response rate, the sample size was adjusted to 426 participants.

Study questionnaire and sampling technique

An electronic questionnaire was utilized as the primary data collection tool. The e-questionnaire was distributed electronically through different platforms (Twitter, WhatsApp, Telegram, emails, and others). In addition to sociodemographic inquiries, 10 questions were posed to test participants’ awareness of glaucoma. A random sampling technique was used to select participants, and then final participants were included based on inclusion criteria and removed based on exclusion criteria.

Ethical considerations

The ethical aspects of this study were overseen by the Scientific Research Ethics Committee, Taif University on September 25, 2023 (reference number: 45-033).

Statistical analysis

Both descriptive and inferential statistical analysis of the data was performed. Simple frequencies and percentages of the sociodemographic characteristics of the participants and their perception, knowledge, and awareness about glaucoma were calculated and tabulated. Awareness was quantified by assigning a value of 1 to correct responses and a value of 0 to incorrect ones. These scores were then summed up, and individuals with more than 50% correct answers were considered to exhibit a substantial level of awareness. Chi-square/Fisher’s exact test was used to determine the association between sociodemographic features and good/poor glaucoma awareness levels. Statistical significance was established at a p-value of 0.05 or less with a 95% confidence interval. All statistical calculations were performed using the SPSS Software version 29.0.0 (IBM Corp., Armonk, NY, USA).

## Results

This study included 1,000 participants. Regarding gender distribution, 58.6% (n = 586) were males. Most participants (n = 630, 63.0%) were in the 18-29-year age group. Regarding education, 51.1% (n = 511) had at least a bachelor’s education, while 44.8% (n = 448) had completed secondary school. The majority (n = 644, 64.4%) were single, and 59.7% (n = 597) were unemployed. Additionally, 90.2% (n = 902) did not have diabetes, and 5.0% (n = 50) did not know whether they had diabetes (Table 1).

**Table 1:** Sociodemographic of the participants assessed for glaucoma awareness (n = 1,000).

Variables		Frequency (n)	Percentage (%)
Gender	Female	414	41.4
	Male	586	58.6
Age (years)	18–29	630	63.0
	30–39	144	14.4
	40–49	152	15.2
	50–59	55	5.5
	+60	19	1.9
Education level	No degree	12	1.2
	Elementary school	29	2.9
	Secondary school	448	44.8
	Under or postgraduate	511	51.1
Marital status	Single	644	64.4
	Married	329	32.9
	Divorced	21	2.1
	Widow	6	0.6
Employment	Employee	403	40.3
	Unemployed	597	59.7
History of diabetes	Don’t know	50	5.0
	No	902	90.2
	Yes	48	4.8

**Figure 1:**
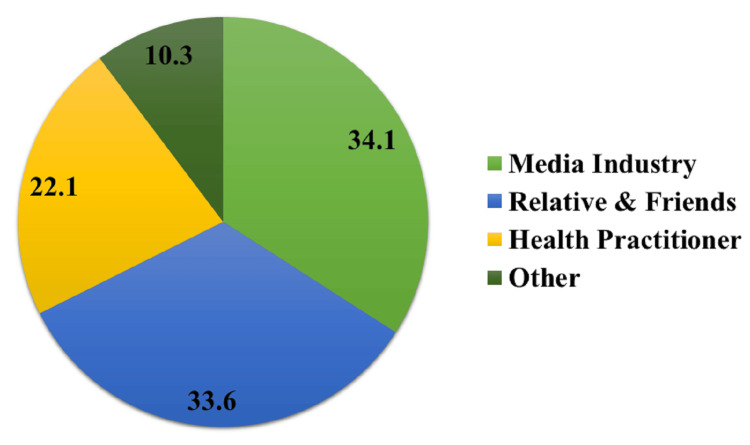
Sources of information about glaucoma.

Participants’ sources of information about glaucoma were diverse. The majority (n = 341, 34.1%) relied on the media industry for information. Around 33.6% (n = 336) obtained information from relatives and friends, while 22.1% (n = 221) consulted health practitioners (Figure 1).

Approximately, 58.5% (n = 585) had heard about glaucoma. Concerning family history, 71.3% (n = 713) reported that none of the family members had glaucoma. Knowledge about normal eye pressure values varied, where 74.2% (n = 742) were unsure. Regarding the definition of glaucoma, 50.4% (n = 504) were uncertain. Symptoms such as progressive vision loss were recognized by 44.1% (n = 441). Participants were divided on whether patients with glaucoma have no symptoms, with 40.1% (n = 401) disagreeing. The majority (n = 631, 63.1%) believed that there was a cure for glaucoma, and 43.1% (n = 431) thought that lost sight could be restored with treatment (Table 2).

**Table 2:** Assessment of knowledge of the different aspects of glaucoma of participants (n = 1,000). *: Correct answers. IOP = intraocular pressure

		Frequency	Percentage
Knowledge about the normal value of eye pressure	Between 11 and 21*	122	12.2
	Between 8 and 12	136	13.6
	I don’t know	742	74.2
Definition of glaucoma	Condition having eye pain	34	3.4
	Condition having nerve damage due to raised IOP	204	20.4
	Condition having nerve damage*	33	3.3
	Condition having raised IOP	225	22.5
	Don’t know	504	50.4
Symptoms of glaucoma	Progressive vision loss*	441	44.1
	Sudden vision loss	64	6.4
	Don’t know	446	44.6
	None of the above	49	4.9
Type of vision loss occurs in glaucoma (n = 505)	Central vision loss	135	13.5
	Peripheral vision loss (tunnel vision)*	222	22.2
	I don't know	148	14.8
Patients with glaucoma have no symptoms (n = 593)	No	401	40.1
	Yes*	192	19.2
Is there any cure for glaucoma (n = 711)	No	80	8.0
	Yes*	631	63.1
Is it possible to restore the lost sight with glaucoma treatment (n = 582)	No*	151	15.1
	Yes	431	43.1

**Figure 2:**
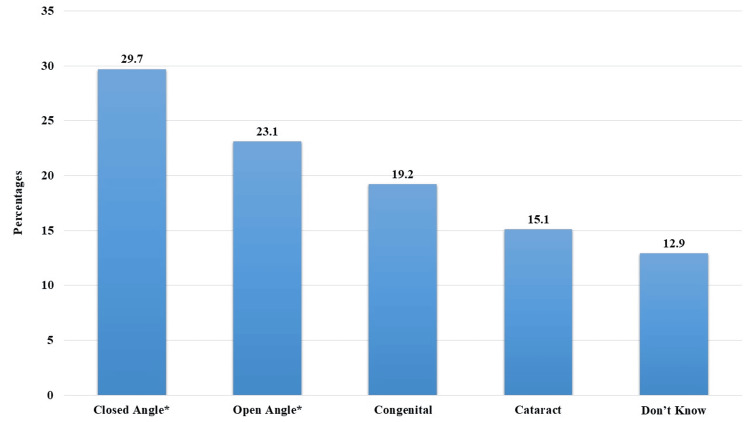
Perception of participants about glaucoma types. *: Correct answer.

Regarding glaucoma types, closed-angle glaucoma (CAG) was perceived by 29.7% (n = 297), while open-angle glaucoma (OAG) was recognized by 23.1% (n = 231). Notably, 12.9% (n = 129) of participants indicated uncertainty regarding the specific glaucoma type (Figure 2).

**Figure 3:**
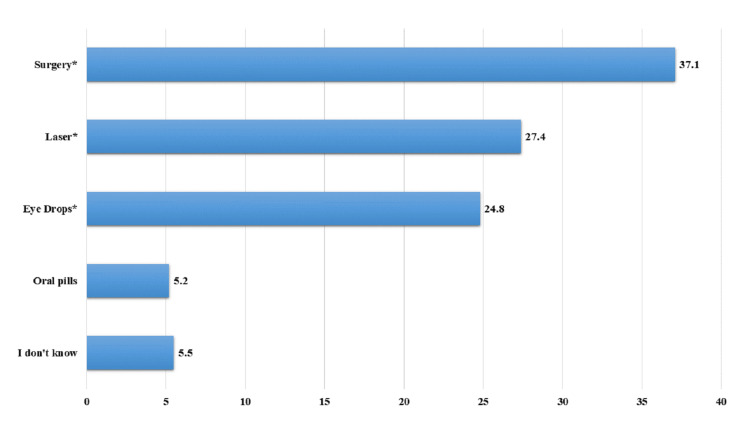
Knowledge of participants about the cure of glaucoma. *: Correct answer.

The majority (n = 371, 37.1%) believed that surgery could cure glaucoma. Laser treatment was perceived as a curative option by 27.4% (n = 274) of participants. Additionally, 24.8% (n = 248) thought eye drops were effective in treating glaucoma (Figure 3).

Regarding age, no statistically significant difference was observed among different age groups with respect to awareness levels (p = 0.362). However, age groups between 40 and 60 years old were the most knowledgeable about the disease. Further, gender showed a strong association (p < 0.001), with women exhibiting higher awareness levels (n = 442, 44.2%) compared to men (n = 322, 32.2%). Educational level displayed a significant association (p = 0.044), with individuals having university or postgraduate degrees demonstrating the highest awareness (n = 413, 41.3%) than those with less education (25% (n = 250) for non-educated, 27.5% (n = 275) for those with elementary education, and 33.4% (n = 334) for those with secondary education). Employment status and marital status did not show significant associations with awareness. Moreover, the presence of diabetes, as a risk factor for glaucoma, did not significantly affect participants’ awareness levels (p = 0.798). Notably, a family history of glaucoma exhibited a substantial association (p < 0.001) with awareness levels. Participants with a positive family history of glaucoma achieved higher awareness scores (n = 450, 45%) in comparison to those without the disease (n = 425, 42.5%) (Table 3).

**Table 3:** Associations of sociodemographic features with the awareness level of glaucoma (n = 1,000).

Variables		Awareness level of glaucoma		P-value
		Poor awareness (n = 628)	High awareness (n = 372)	
		Age (years)	18–29		405	225	0.362
30–39	90		54				
40–49	89		63				
50–59	30		25				
+60	14		5				
Gender	Female	231	183	<0.001
	Male	397	189	
Educational level	No degree	9	3	0.044
	Elementary school	21	8	
	Secondary school	298	150	
	Under or postgraduate	300	211	
Employment status	Employee	245	158	0.281
	Unemployed	383	214	
Marital status	Single	413	231	0.667
	Married	198	131	
	Divorced	13	8	
	Widow	4	2	
Suffer from diabetes	Yes	28	20	0.798
Family history of glaucoma	Yes	84	69	<0.001

**Figure 4:**
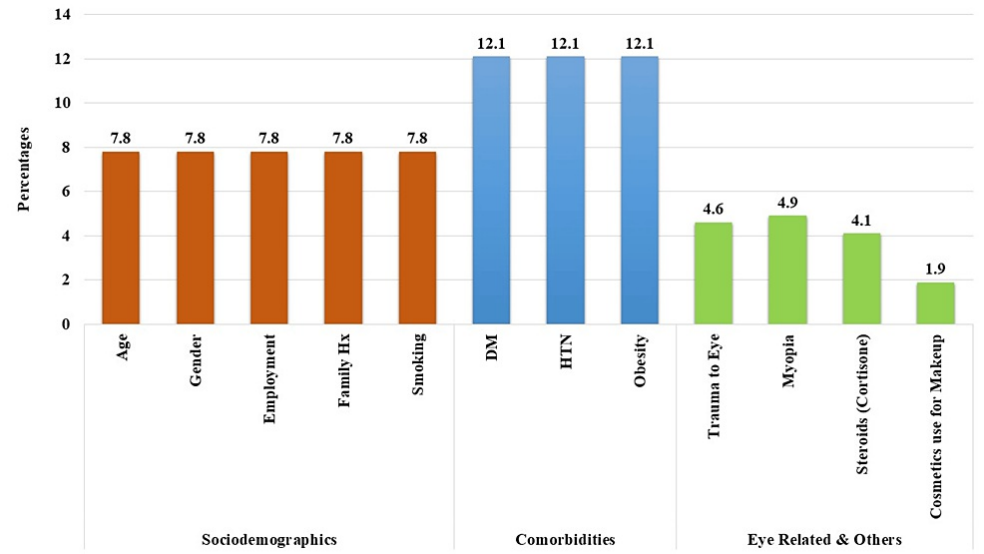
Perception of participants about the factors that increase the risk of glaucoma. Correct answer: All the above factors can increase the risk for glaucoma except for the cosmetic use of makeup. Hx = history; DM = diabetes mellitus; HTN = hypertension

Sociodemographic factors such as age, gender, employment, family history, and smoking received 7.8% (n = 78) each. Comorbidities such as diabetes, hypertension, and obesity were noted by 12.1% (n = 121). Eye-related and other factors such as trauma, myopia, steroids, and cosmetics use received lower attention (Figure 4).

## Discussion

Glaucoma, a silent but devastating eye disease, poses a substantial threat to global eye health [2]. Its multifactorial etiology encompasses variables such as genetics, age, intraocular pressure, family history, and race [3]. Swift diagnosis, timely intervention, and effective management stand as pivotal factors in mitigating irreversible vision loss caused by this insidious condition [4]. This study aimed to provide valuable insights into the awareness levels and knowledge about glaucoma among the people living in Taif City, Saudi Arabia.

Sociodemographic data revealed interesting patterns. The majority of women showed high awareness, suggesting that females exhibited a higher awareness of glaucoma compared to males [15]. This finding emphasizes the need for targeted awareness of glaucoma among the Taif City population.

As the 18-29-year age group had the highest representation in glaucoma awareness assessment, it is essential to note that glaucoma primarily affects older individuals due to age-related risk. Awareness efforts should encompass all age groups, as even the young might be at risk and need further education [19]. Thus, glaucoma awareness campaigns should be designed to reach individuals across all age groups.

Education plays a crucial role in awareness levels as 51.1% (n = 511) of participants had under or postgraduate education, while 44.8% (n = 448) had completed secondary school. Interestingly, the educational level did show a significant association with awareness (p = 0.044). Individuals with no education or those with elementary school degrees exhibited poorer awareness compared to those with secondary school or higher education [20].

Understanding the source of participants’ information about glaucoma is essential for tailoring awareness campaigns. Most participants relied on the media industry for information, followed closely by those who obtained information from relatives and friends [21]. This emphasizes the need for a diverse strategy in spreading glaucoma awareness, including media, social conversations, and healthcare professional involvement in education.

Regarding awareness level, 58.5% (n = 585) of participants were aware of glaucoma, indicating that a substantial proportion were unaware of the disease. To raise awareness, targeting those who are completely unaware is crucial [22].

Although family history is a significant glaucoma risk factor, 71.3% (n = 713) reported no family history of glaucoma. This underscores the importance of understanding one’s family medical history and the need for regular eye examinations [23,24].

Knowledge about normal eye pressure values was limited, with 74.2% (n = 742) of unsure participants. This lack of knowledge can lead to misconceptions about glaucoma, which is often associated with damage to the optic nerve. Education should emphasize that glaucoma has more than one type and can occur even with normal eye pressure (OAG).

It is crucial to understand that glaucoma is symptomless until significant damage occurs. This study found that 44.1% of participants recognized symptoms such as progressive vision loss [25,26]. However, there was uncertainty among 40.1% about whether glaucoma patients have symptoms or not. This highlights the need to educate the public about the “silent thief of sight” nature of glaucoma, where symptoms might not be apparent until irreversible damage occurs.

The majority (n = 631, 63.1%) believed glaucoma had a cure, and 43.1% (n = 431) thought lost sight could be restored. However, these optimistic beliefs might not align with reality. Education should stress early detection and management, although full vision restoration is not always guaranteed.

Understanding participant perceptions of glaucoma types with 29.7% (n = 297) with CAG, 23.1% (n = 231) OAG, 19.2% (n = 192) congenital, 15.1% (n = 151) cataract causes, and 12.9% (n = 129) were uncertain [27]. These findings underscore the need for clear and concise education about the different types of glaucoma to dispel misconceptions.

Participants’ knowledge about potential cures for glaucoma is also noteworthy. The majority (n = 371, 37.1%) believed that surgery could cure glaucoma, while 27.4% (n = 274) perceived laser treatment as a curative option [28]. Education should clarify that while these treatments can help manage glaucoma, there is no definitive cure, and ongoing monitoring is essential.

Finally, the associations between sociodemographic characteristics and glaucoma awareness reveal that gender showed a strong association, with women exhibiting higher awareness. A positive family history of glaucoma exhibited a significant association with awareness. This finding highlights the importance of targeting individuals with a family history of the disease for education and regular eye screenings.

The main limitation of this study is the potential selection bias as it focuses on a specific region (Taif City, Saudi Arabia) and might not represent broader populations. Moreover, the study used self-reported data susceptible to information bias. Its cross-sectional design hinders causal inference and might miss changes in awareness over time. It also lacked exploration of reasons behind observed associations, impacting future evidence-based action plans.

## Conclusions

This study revealed glaucoma awareness among adults in Taif City. Gender, education, and family history played significant roles in shaping awareness levels. There is a need for targeted educational efforts to improve knowledge about glaucoma in the community. This study underscores the importance of tailored glaucoma awareness campaigns, especially targeting men, less-educated individuals, and those lacking awareness about glaucoma. It emphasizes the crucial role of disseminating accurate information. Leveraging family history as a risk factor for targeted education and early detection is vital. These findings have significant implications for informing public health initiatives aimed at raising glaucoma awareness, focusing on old age and less-educated individuals, dispelling misconceptions, and highlighting the “silent” nature of the disease in the region.
